# Supplementary figures and images for: A spatially uniform illumination source for widefield multi-spectral optical microscopy
Source: PLoS One. 2023 Oct 18;18(10):e0286988. doi: 10.1371/journal.pone.0286988 (PMC10584126; doi:10.1371/journal.pone.0286988)

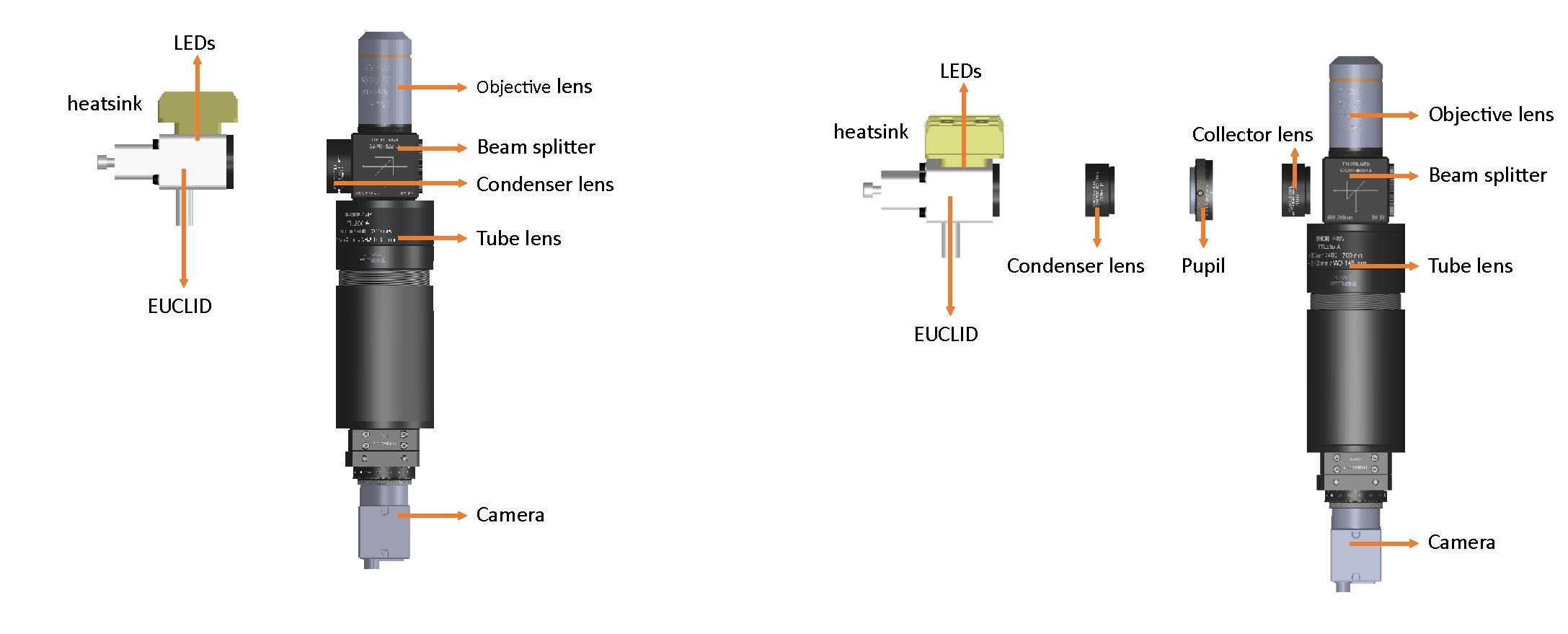

Supplement: S1 Fig — Detailed schematics of the imaging setups. Left: with critical illumination, Right: Koehler illumination. The light integration devices (LIDs) or direct LED dies were aligned to the imaging optics in identical conditions to acquire field profiles. (TIF) [file pone.0286988.s001.tif]

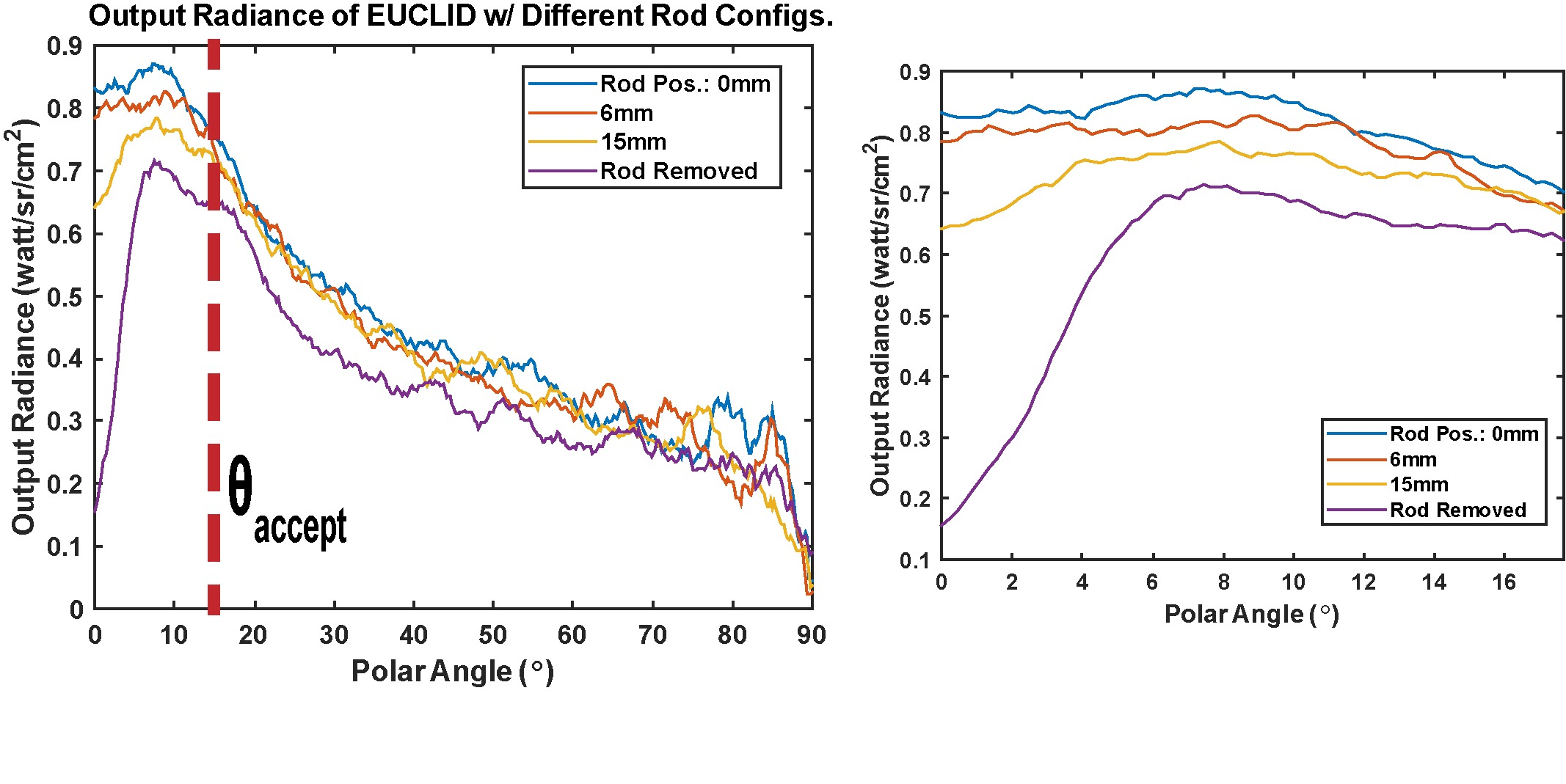

Supplement: S2 Fig — Output Radiance cross sections of EUCLID with 5 mm output port and rod diameter when rod is positioned different locations. Left: Output radiance for all polar angles. Acceptance angle is defined by lens 3 in Fig 3. Right: Zoomed section of left graph for angles that lies within the acceptance angle. (TIF) [file pone.0286988.s002.tif]

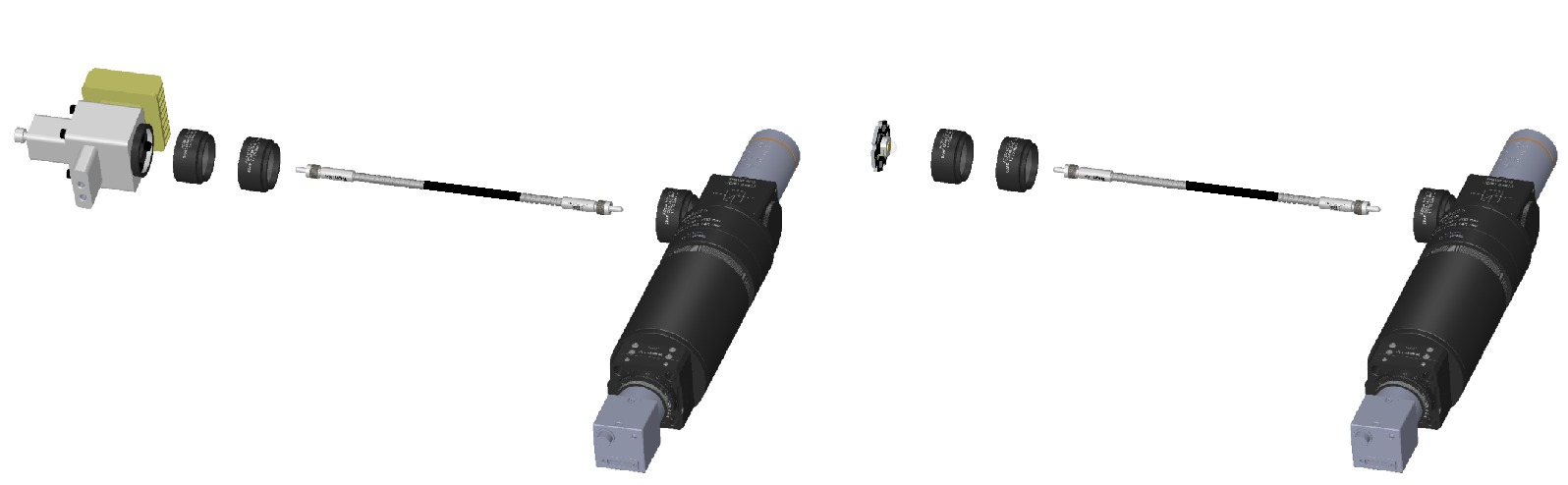

Supplement: S3 Fig — Schematic of the imaging setups for fiber alignment, with direct LEDs (right) and with EUCLID (left). The light output of direct LED dies and the EUCLID were coupled to the fiber tip by a lens pair for demagnification. The output of the fiber tip is then imaged by using the same collection optics as previous setups. (TIF) [file pone.0286988.s003.tif]

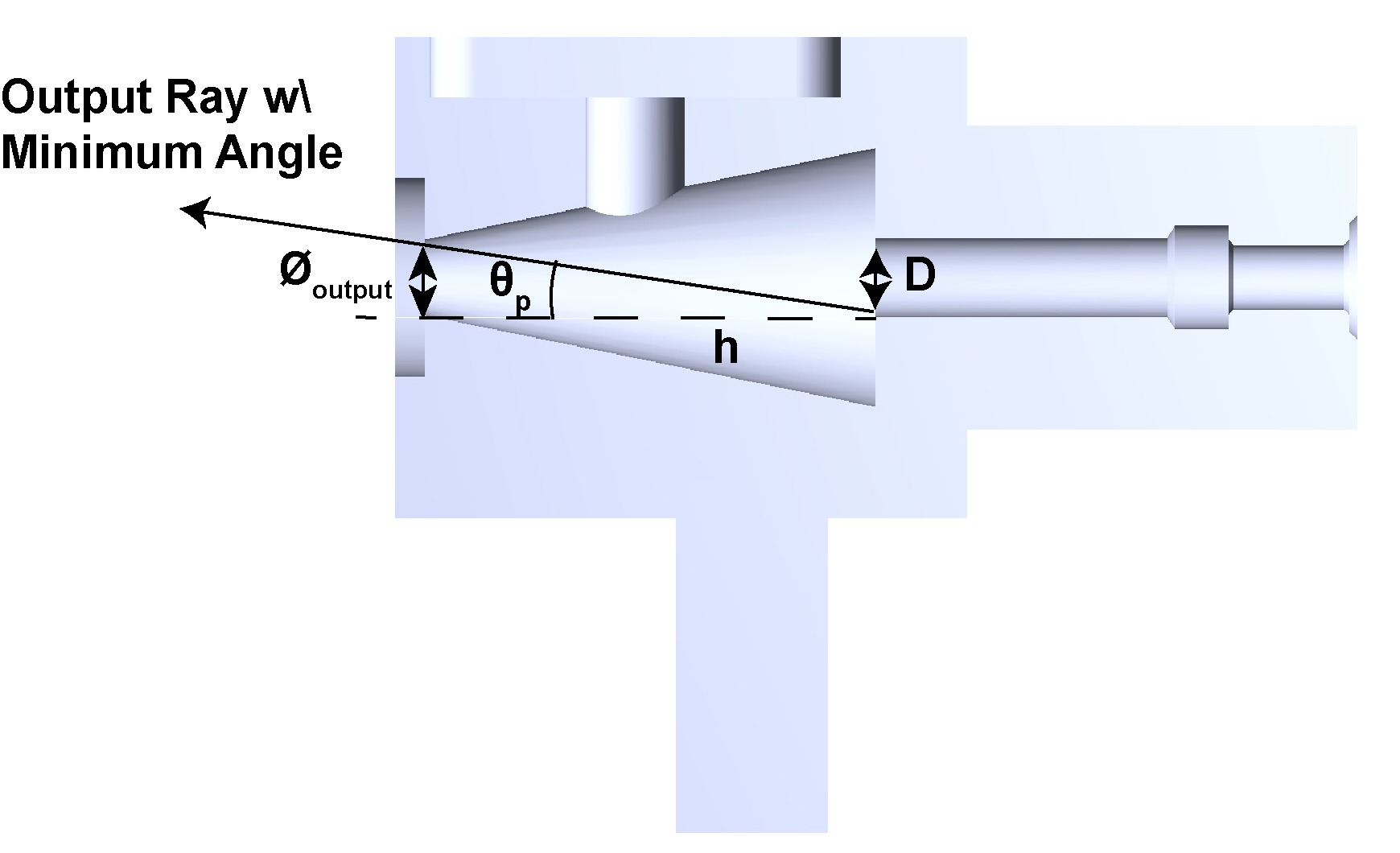

Supplement: S4 Fig — Toy picture of the EUCLID geometry where the output ray with the minimum exit angle is indicated. h is the height, ⌀output is the output port and D is the guiding hole diameter of the EUCLID. (TIF) [file pone.0286988.s004.tif]

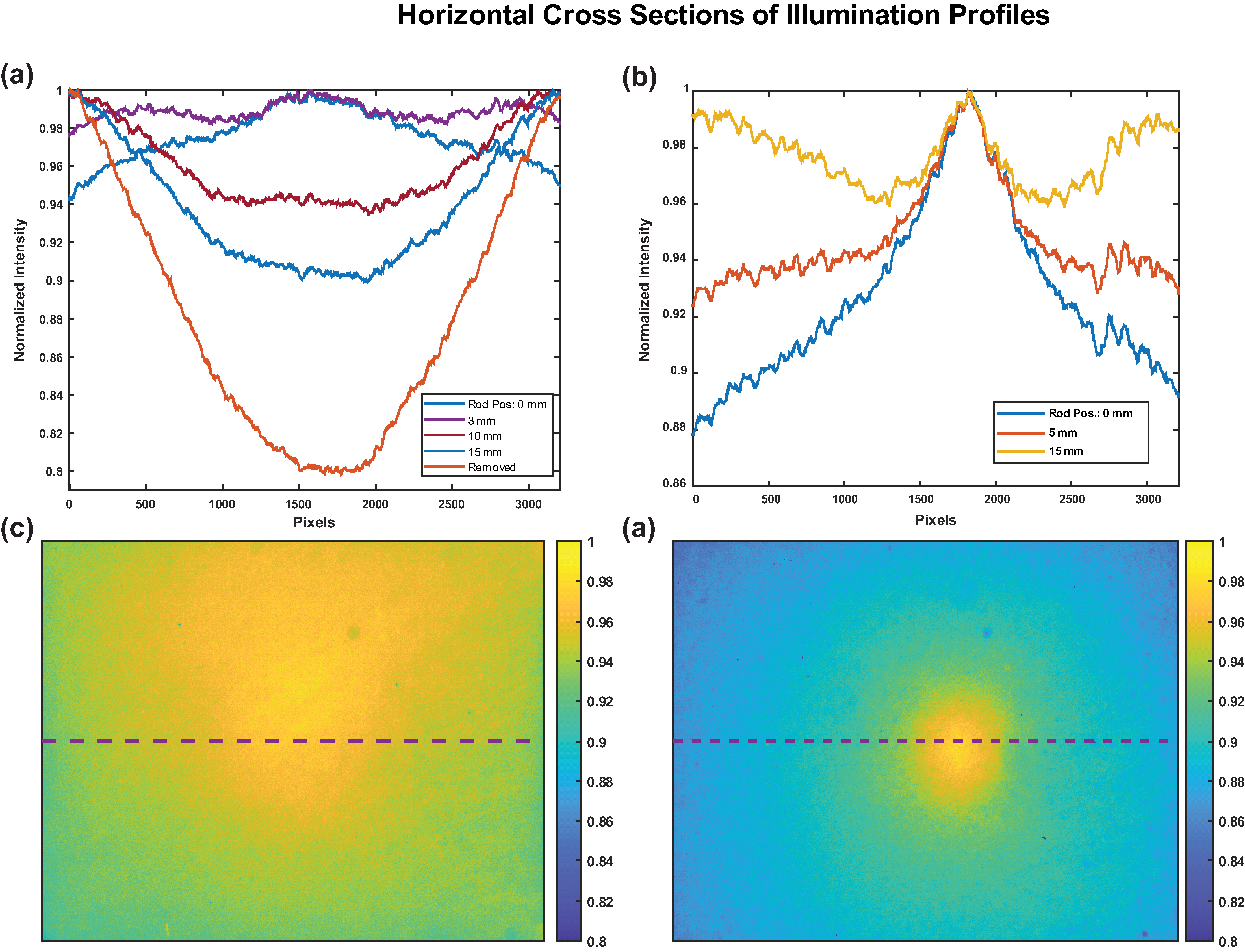

Supplement: S5 Fig — Horizontal cross sections of two different non-optimized EUCLID in different illumination configurations. EUCLID with rod diameter 1/4” (a,c) is tested under critical illumination configuration. Normalized heatmap (c) and corresponding horizontal cross sections for different rod positions (a) are indicated. EUCLID with rod diameter 3/16” (b,d) is tested under Koehler illumination configuration. Normalized heatmap (d) and corresponding horizontal cross sections for different rod positions (b) are indicated. (TIF) [file pone.0286988.s005.tif]
