# Supplementary material for: A spatially uniform illumination source for widefield multi-spectral optical microscopy
Source: PLoS One. 2023 Oct 18;18(10):e0286988. doi: 10.1371/journal.pone.0286988 (PMC10584126; doi:10.1371/journal.pone.0286988)
Supplement: S1 Table — First two metrics in the table, plateau uniformity and flatness factor, is defined in the ISO 13694:2000 standard [23] for continuous waves. The metric values, expect EUCLID’s and IS’s, is taken from corresponding references [20, 21, 24]. The ISO values for [18–20] is characterized without the effect of the external optical element, such as objective and tube lenses, dichorics or beam splitters, etc. The commercial refractive elements, TopShape and PiShaper, also require spatial filtering of the input beam. The values for the remaining are calculated in a common epi-illumation system, where the effects of the external components are considered. Performance metrics of different flat-top illumination system, where the effects of the external components are considered. (PDF) [file pone.0286988.s006.pdf]

S1 Table. Comparison of Different Flat-Fielding Systems First two metrics in the table, plateau uniformity and flatness factor, is defined in the ISO 13694:2000 standard [23] for continuous waves. The metric values, expect EUCLID's and IS's, is taken from corresponding references [20, 21, 24]. The ISO values for [18–20] is characterized without the effect of the external optical element, such as objective and tube lenses, dichorics or beam splitters, etc. The commercial refractive elements, TopShape and PiShaper, also require spatial filtering of the input beam. The values for the remaining are calculated in a common epi-illumation system, where the effects of the external components are considered. Performance metrics of different flat-top illumination systems. $: $<$ 1k $, $$: 1k-3k $, $$$: $>$ 5k $

System	**EUCLID**	**Kohler Integrator [20,24]**	**PiShaper [19]**	**TopShape [18]**	**Deschamps et. al. [21]**	**Coumans et. al. [9]**
Plateau Uniformity	0.0180	0.0621	0.0899	0.0996	N/A	N/A
Flatness Factor	0.9648	0.8442	0.7348	0.6725	N/A	N/A
STD to Mean Rat.	0.0083	N/A	N/A	N/A	0.0250	0.015-0.012
Source	LED	Laser	Laser	Laser	Laser	Halogene Lamp
Cost	$	$$	$$$	$$$	$$	$$
