# Supplementary material for: A spatially uniform illumination source for widefield multi-spectral optical microscopy
Source: PLoS One. 2023 Oct 18;18(10):e0286988. doi: 10.1371/journal.pone.0286988 (PMC10584126; doi:10.1371/journal.pone.0286988)
Supplement: S1 Appendix — To quantify the illumination profile uniformity, we have defined uniformity regions as the largest area enclosed, that yield a normalized intensity deviation under 1% or 0.5%. We calculated the radii of the regions with a custom MATLAB algorithm. The algorithm initializes the ROI with a circular region (radius of 25 pixels) and compares the mean values of the current ROI to the annulus (with a thickness of 10 pixels) just outside of the initial region. If the average absolute brightness deviation of the annulus, compared to the previous ROI, is within the specified range (1 or 0.5%) the algorithm continues to evaluate the absolute brightness deviation in the next annulus with the same thickness, just outside of the previous annular ROI. The algorithm iterates until the specified annular ring indicates that the uniformity level has decreased below the specified range or the radii hits the corner of the FOV. The uniformity regions for all sources are illustrated in Fig 4 and the calculated radii of 1% and 0.5% uniformity regions are given in Table 2 in the manuscript. We also applied Gaussian filter (σ = 5) to remove the small artefacts on the sample, such as dust particles, etc. (PDF) [file pone.0286988.s007.pdf]

S1 Appendix. Custom MATLAB Code to Determine Uniformity Regions. To quantify the illumination profile uniformity, we have defined uniformity regions as the largest area enclosed, that yield a normalized intensity deviation under 1% or 0.5%. We calculated the radii of the regions with a custom MATLAB algorithm. The algorithm initializes the ROI with a circular region (radius of 25 pixels) and compares the mean values of the current ROI to the annulus (with a thickness of 10 pixels) just outside of the initial region. If the average absolute brightness deviation of the annulus, compared to the previous ROI, is within the specified range (1 or 0.5%) the algorithm continues to evaluate the absolute brightness deviation in the next annulus with the same thickness, just outside of the previous annular ROI. The algorithm iterates until the specified annular ring indicates that the uniformity level has decreased below the specified range or the radii hits the corner of the FOV. The uniformity regions for all sources are illustrated in Fig.4 and the calculated radii of 1% and 0.5% uniformity regions are given in Table 2 in the manuscript. We also applied Gaussian filter ($\sigma = 5$) to remove the small artefacts on the sample, such as dust particles, etc.
