# Supplementary material for: A spatially uniform illumination source for widefield multi-spectral optical microscopy
Source: PLoS One. 2023 Oct 18;18(10):e0286988. doi: 10.1371/journal.pone.0286988 (PMC10584126; doi:10.1371/journal.pone.0286988)
Supplement: S2 Appendix — (PDF) [file pone.0286988.s008.pdf]

S2 Appendix. Geometric Optics Approach to Determine Optimal EUCLID Parameters. We designed EUCLID to control illumination profile for epi-illumination brightfield microscopes and tested two different illumination configuration, critical and Koehler. In Koehler configuration, the output of the EUCLID is imaged on the back focal plane of the imaging objective and defocused on the sample. An output ray whose height and angle are r and θ, can be ray-traced to the sample plane as,

$$\begin{bmatrix} r_{sample} \\ \theta_{sample} \end{bmatrix} = \begin{bmatrix} 1 & f \\ 0 & 1 \end{bmatrix} \begin{bmatrix} 1 & 0 \\ -\frac{1}{f} & 1 \end{bmatrix} \begin{bmatrix} 1 & f \\ 0 & 1 \end{bmatrix} \begin{bmatrix} r \\ \theta \end{bmatrix} \tag{3}$$

The pass angle, given in Eq.2, determines the maximum area that can be altered by the movable rod, whose radius can be found as,

$$r_{max} = f\theta_p \tag{4}$$

In critical illumination, it is preferable to match the output dimension and the rod diameter for small aperture systems, since it gives the flexibility to adjust the distribution of the field profile with changing the rod position while minimizing the power loss due to the hole.
